# Transcriptome Analysis of Chlorantraniliprole Resistance Development in the Diamondback Moth *Plutella xylostella*

**DOI:** 10.1371/journal.pone.0072314

**Published:** 2013-08-20

**Authors:** Qingsheng Lin, Fengliang Jin, Zhendi Hu, Huanyu Chen, Fei Yin, Zhenyu Li, Xiaolin Dong, Deyong Zhang, Shunxiang Ren, Xia Feng

**Affiliations:** 1 Insititute of Plant Protection, Guangdong Academy of Agricultural Sciences, Guangzhou, P. R. China; 2 Engineering Research Center of Biological Control, Ministry of Education, South China Agricultural University (SCAU), Guangzhou, P. R. China; 3 Guangdong Provincial Key Laboratory of High Technology for Plant Protection, Guangzhou, P. R. China; Swedish University of Agricultural Sciences, Sweden

## Abstract

**Background:**

The diamondback moth *Plutella xyllostella* has developed a high level of resistance to the latest insecticide chlorantraniliprole. A better understanding of *P. xylostella*’s resistance mechanism to chlorantraniliprole is needed to develop effective approaches for insecticide resistance management.

**Principal Findings:**

To provide a comprehensive insight into the resistance mechanisms of *P. xylostella* to chlorantraniliprole, transcriptome assembly and tag-based digital gene expression (DGE) system were performed using Illumina HiSeq™ 2000. The transcriptome analysis of the susceptible strain (SS) provided 45,231 unigenes (with the size ranging from 200 bp to 13,799 bp), which would be efficient for analyzing the differences in different chlorantraniliprole-resistant *P. xylostella* stains. DGE analysis indicated that a total of 1215 genes (189 up-regulated and 1026 down-regulated) were gradient differentially expressed among the susceptible strain (SS) and different chlorantraniliprole-resistant *P. xylostella* strains, including low-level resistance (GXA), moderate resistance (LZA) and high resistance strains (HZA). A detailed analysis of gradient differentially expressed genes elucidated the existence of a phase-dependent divergence of biological investment at the molecular level. The genes related to insecticide resistance, such as P450, GST, the ryanodine receptor, and connectin, had different expression profiles in the different chlorantraniliprole-resistant DGE libraries, suggesting that the genes related to insecticide resistance are involved in *P. xylostella* resistance development against chlorantraniliprole. To confirm the results from the DGE, the expressional profiles of 4 genes related to insecticide resistance were further validated by qRT-PCR analysis.

**Conclusions:**

The obtained transcriptome information provides large gene resources available for further studying the resistance development of *P. xylostella* to pesticides. The DGE data provide comprehensive insights into the gene expression profiles of the different chlorantraniliprole-resistant stains. These genes are specifically related to insecticide resistance, with different expressional profiles facilitating the study of the role of each gene in chlorantraniliprole resistance development.

## Introduction

The diamondback moth (DBM) *Plutella xyllostella* (L.) (Lepidoptera: Plutellidae), an oligophagous pest feeding only on the plant family Brassicaceae, is one of the most widely distributed insects in the world [1]. Currently, this moth has been reported from more than 80 countries and is known to lead to severe losses of cruciferous vegetables and rapeseed crops [2]. *P. xylostella* can lead to an up to 52% loss of the market yield of cabbage, and the total cost associated with *P. xylostella* management is from US$4 billion to US$5 billion per year [3], [4]. Its harmful effects are predominantly due to its strong ability to develop insecticide resistance. To date, *P. xylostella* has developed resistance to almost all classes of insecticides, including organochlorines, organophosphates, carbamates, pyrethroids, insect growth regulators, abamectins, pyrazoles, oxadiazines, neonicotinoids, and *Bacillus thuringiensis* [5]–[8]. Moreover, certain populations of *P. xylostella* have also developed resistance to some new active ingredients, such as chlorantraniliprole in South China and other countries in southeast Asia [9], [10].

Chlorantraniliprole (Rynaxypyr) is a new chemical insecticide that belongs to the chemical class anthranilic diamides [11]. As the first member of the anthranilic diamides, chlorantraniliprole has a novel mode of action as an activator of insect ryanodine receptors, which can lead to insect feeding cessation, lethargy, muscle paralysis, and ultimately death by binding to ryanodine receptors and activating the uncontrolled release of calcium stores [12]–[15]. This mode of action is different from other classes of insecticides. Therefore, there is no cross-resistance between chlorantraniliprole and other groups of insecticides [16]. In addition, its high insecticidal potency, relatively low toxicity to beneficial arthropods and high degree of mammalian safety make it a perfect fit for integrated pest management (IPM) programs [15]. Chlorantraniliprole has currently been proven to be the most active compound against lepidopteran pests [16]. It was registered for use against lepidopteran pests in southeast Asia in May 2007 and in China in May 2008 [11]. By 2011, it had been registered for use in more than 80 countries, and the turn-over of chlorantraniliprole-based brands alone reached over $500 million in 2011, placing it among the 5 top-selling insecticides worldwide [17]. However, *P. xylostella* has displayed a strong ability for rapid resistance development to chlorantraniliprole. Just two years after its application in 2011, a high level of resistance by *P. xylostella* was reported (resistance factor >600-fold) in Guangdong, China, which led to the outbreak of *P. xylostella* populations that produced a significant loss to the vegetable industry in the area [18]. It has recently been reported that the *P. xylostella* from southern China displays a high level of resistance to chlorantraniliprole, whereas the *P. xylostella* from central and northern China possess low and moderate levels of resistance to chlorantraniliprole, indicating that the resistance of *P. xylostella* to chlorantraniliprole has spread from southern to northern China [9]. Therefore, there is an urgent need for a better treatment measure to stop the development and spread of the resistance of *P. xylostella* to chlorantraniliprole. Research on insecticide resistance mechanisms is an important step to gain knowledge for the management of insecticide resistance, which will allow us to identify a more effective manner to monitor and manage insecticide resistance [19]. In this study, we investigated the factors involved in chlorantraniliprole resistance (CR) to provide appropriate measures for the IPM control of *P. xylostella*.

Resistance may alter target sites and increase the rate of metabolism. In the past, studies on the resistance mechanisms of *P. xylostella* were predominantly focused on the detoxification activities resulting from metabolism and the cloned ryanodine receptor genes. However, little is known about the dynamic expression of genes related to its pesticide resistance, and consequently, much less information is available for us to understand the molecular mechanisms of chlorantraniliprole resistance in *P. xylostella*. During the past few years, next-generation sequencing technologies have provided effective tools for high-throughput sequencing, which has improved the efficiency and speed of gene discovery. The Illumina sequencing technology in particular, which enables the generation of over one billion bases of high-quality DNA sequence per run at less than 1% of the cost of capillary-based methods, has made it possible to perform genomic research on insects. A large number of genes associated with specific developmental stages and insecticides have been identified from the oriental fruit fly [*Bactrocera dorsalis* (Hendel)], the migratory locust [*Locusta migratoria manilensis* (Mayen)], the tobacco whitefly [*Bemisia tabaci* (Gennadius)], the citrus red mite [*Panonychus citri* (McGregor)] and *P. xylostella* by *de novo* assembly [20]–[24].

In this study, to obtain a better understanding of CR in *P. xylostella*, the transcriptome of a susceptible strain (SS) of *P. xylostella* was sequenced using a high-throughput sequencing platform (Illumina HiSeq™ 2000). Unigenes (45,231) with an average length of 532 bp were obtained, and hundreds of insecticide targets and metabolism genes were identified. Furthermore, to obtain detailed genetic information for CR mechanism, the gene expression profiles for four *P. xylostella* populations with four levels of CR (sensitive, low level resistance, moderate resistance, and high resistance) were analyzed using a digital gene expression system (RNA-Seq analysis), and the unigenes or pathways associated with CR in *P. xylostella* were identified. The assembled, annotated transcriptome sequences and gene expression profiles will provide a valuable genetic resource for further understanding the molecular resistance mechanisms of *P. xylostella* against chlorantraniliprole.

## Results

### Illumina sequencing and read assembly

To obtain an overview of the *P. xylostella* gene expression profile of a SS strain, a cDNA library derived from whole bodies of a *P. xylostella* SS strain at eight different developmental stages (egg, 1^st^, 2^nd^, 3^rd^ and 4^th^ instar larvae, prepupa and pupa, and adult) was constructed using Illumina HiSeq™ 2000. Reads (27,337,100) and clean nucleotides (2,460,339,000) were obtained and assembled into 105,298 contigs with a mean length of 284 bp. Finally, the contigs were connected using Trinity software to produce unigenes, with 45,231 unigenes obtained. The mean size of these unigenes was 532 bp and the lengths ranged from 200 to 13,799 bp with more than 4,700 larger than 1,000 bp (Table 1).

**Table 1:** Summary of the *P. xylostella* transcriptome.

Total number of reads	27,337,100
Total clean nucleotides	2,460,339,000
Total number of contigs	105,298
Mean length of contigs	284
Total number of unigenes	45,231
Mean length of a unigene	532
Sequences with E-values <10-5	27,516

### Annotation of predicted proteins

For annotation, distinct gene sequences were blasted against the non-redundant (nr) NCBI protein database using BLASTx with a cut-off E-value of 10^−5^. There were a total 27,516 unigenes (60.83%) matched with known genes (Table S1). The E-value distribution of the hits in the nr database indicated that 22.4% of the mapped sequences were smaller than 1.0E-60 and had strong homologies, whereas the other homologous sequences had values that ranged from 1.0E-5 to 1.0E-60 (Fig. S1 A). Approximately 13.9% of the unigenes shared 80% or higher similarity distributions, and 86.1% of the hits had a similarity ranging from 15% to 80% (Fig. S1 B). For the species’ distribution, 25.06% of the unigenes were matched to sequences from the monarch butterfly *Danaus plexippus* (L.), followed by the red flour beetle *Tribolium castaneum* (Herbst) (13.13%), silkworm *Bombyx mori* (L.) (6.98%), the gambia anopheles (*Anopheles gambiae* Giles str. PEST) (4.75%), *Nasonia vitripennis* (Walker) (3.75%), *Harpegnathos saltator* (Jerdon) (2.96%), and *Acromyrmex echinatior* (Forel) (2.94%). The other sequences, which composed up to 40.43%, had hits with other insect species, such as *Lepeophtheirus salmonis* (Krøyer), *Acyrthosiphon pisum* (Harris) and *Drosophila virilis* (Sturtevant) (Fig. S1 C).

### Gene ontology (GO) and clusters of orthologous groups’ (COG) classifications

**Figure 1:**
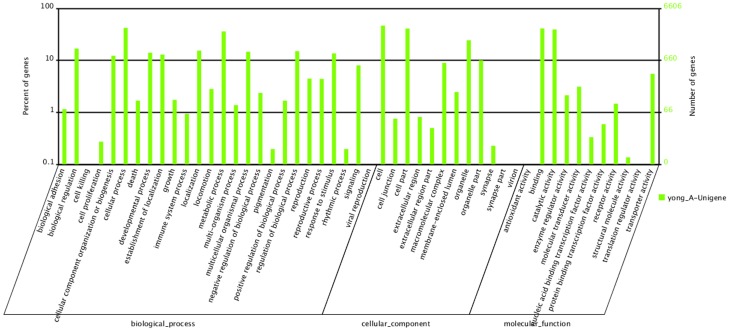
Histogram presentation of the gene ontology classification. The right y-axis indicates the number of genes in a category, whereas the left y-axis indicates the percentage of a specific category of genes in that main category.

Gene ontology (GO) and clusters of orthologous groups (COG) were used to predict and identify the possible functions for the annotated sequences of the SS strain. For the GO analysis, 6,606 unigenes were categorized into 49 functional groups (Fig. 1). The “cellular process”, “cell” and “binding” terms contained 2,786, 3,081 and 2,719 unigenes, respectively, and were dominant in each of the three main categories (biological process, cellular component, and molecular function). Only one unigene was predicted to act in the “cell killing and tow in the virion”. The functions of some genes involved in the metabolic process, immune system process, enzyme regulator activity, receptor activity and catalytic activity terms can be related to CR development in *P. xylostella*.

**Figure 2:**
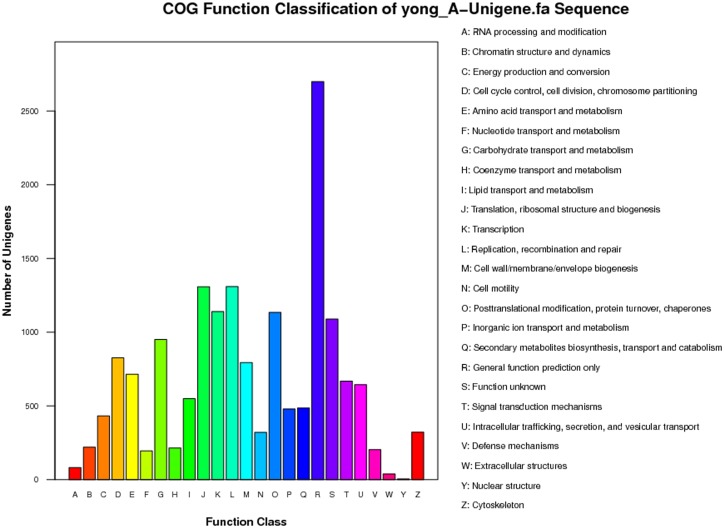
Histogram presentation of clusters of orthologous groups’ (COG) classifications.

COG is a database where orthologous gene products are classified. To further improve the annotation of our transcriptome library, the sequences of the unigenes were aligned in the COG database to predict and identify their possible functions. According to sequence homology, a total of 7,815 sequences were annotated in the COG database and could be divided into 26 specific categories. Among the 26 COG categories, the cluster ‘general function prediction only’ was the largest group, containing 2,699 unigenes, followed by ‘replication, recombination and repair’ (1,309, 7.79%), ‘translation, ribosomal structure and biogenesis’ (1,308, 7.78%), ‘transcription’ (1,139, 6.77%) ‘posttranslational modification, protein turnover, chaperones’ (1,134, 6.74%) and function unknown (1,089, 6.48%). Other clusters contained less than 1,000 sequences, especially ‘nuclear structure’, with only 4 unigenes (0.024%), representing the smallest groups (Fig. 2).

The kyoto encyclopedia of genes and genomes (KEGG) is a database that integrates genomic, chemical and systemic functional information. Using KEGG, we were able to obtain information for our data regarding the pathways and gene functions that are associated with the CR molecular mechanism in *P. xylostella*. In total, 17,140 sequences from our transcriptome were assigned to 255 KEGG pathways. The gene variation in the sequences of the following pathways were observed: ‘metabolic pathways’ (2,798, 16.32%), ‘pyrimidine metabolism’ (372, 2.17%), ‘vascular smooth muscle contraction’ (316, 1.84%), ‘drug metabolism- other enzymes’ (246, 1.44%), ‘calcium signaling pathway’ (245, 1.43%), ‘wnt signaling pathway’ (241, 1.41%), ‘glycerolipid metabolism’ (205, 1.2%), ‘drug metabolism-cytochrome P450’ (164, 0.96%), ‘cardiac muscle contraction’ (162, 0.95%), ‘metabolism of xenobiotics by cytochrome P450’ (155, 0.9%), ‘GABAergic synapse’ (119, 0.69%), ‘nicotinate and nicotinamide metabolism’ (57, 0.33%) and ‘caffeine metabolism’ (36, 0.21%). These changes may be associated with the development and levels of CR in *P. xylostella* (Table S2).

### Genes associated with the insecticide targets and their related metabolism

As we were interested in finding the molecular mechanism of CR in *P. xylostella*, the sequences related to insecticide targets and metabolism were analyzed and compared with the sequences from NCBI nucleotides and the EST database. As shown in Table 2, a number of sequences related to the insecticide’s metabolism and its targets, such as cytochrome P450, carboxylesterase (CaE), glutathione *S*-transferase (GST), superoxide dismutase (SOD), prophenoloxidase (PPO), insecticide targets, the gamma-aminobutyric acid receptor, sodium channel and ryanodine receptor, were analyzed. Among them, the ryanodine receptor (RyR) was a target of chlorantraniliprole. In total, 16 ryanodine receptor-related sequences were obtained (Table 3). These annotations provide a valuable resource for investigating specific processes, functions and pathways during studies of the resistance molecular mechanisms to chlorantraniliprole in *P. xylostella*.

**Table 2:** Genes related to the insecticide targets and metabolism.

Gene name	NnD	NpD
Carboxylesterase	96	17
Glutathione *S*-transferase	61	21
NADH dehydrogenase	60	25
Cytochrome P450	156	28
Acetylcholinesterase (AChE)	14	5
Superoxide dismutase	19	7
Prophenoloxidase	16	9
Gamma-aminobutyric acid receptor	47	43
Nicotinic acetylcholine receptor (nAChRs)	19	2
Sodium channel	15	11
Ryanodine receptor	16	14

NnD: Number of sequences with a hit in the nr database; NpD: Number of known sequences for *P. xylostella* from the NCBI nucleotide database.

**Table 3:** Identified ryanodine receptor genes.

Gene ID	Gene Length	Nr-ID	Nr-Evalue	Nr-annotation
CL253.Contig1_yong_A	1,841	gi|354463161|gb|AER25355.1|	0	*P. xylostella*
CL2655.Contig1_yong_A	642	gi|356470637|gb|AET09964.1|	1.00E-120	*P. xylostella*
CL2655.Contig2_yong_A	4,184	gi|354463159|gb|AER25354.1|	0	*P. xylostella*
CL3354.Contig1_yong_A	419	gi|332025910|gb|EGI66066.1|	3.00E-15	*Acromyrmex echinatior*
CL5169.Contig1_yong_A	1,666	gi|356470637|gb|AET09964.1|	0	*P. xylostella*
CL4637.Contig1_yong_A	1,991	gi|356470637|gb|AET09964.1|	0	*P. xylostella*
CL5966.Contig1_yong_A	1,186	gi|356470637|gb|AET09964.1|	0	*P. xylostella*
CL6021.Contig1_yong_A	1,094	gi|118795372|ref|XP_561090.4|	5.00E-17	*Anopheles gambiae* str. PEST
CL7443.Contig1_yong_A	557	—	—	—
Unigene17929_yong_A	392	—	—	—
Unigene17930_yong_A	260	—	—	—
Unigene2391_yong_A	315	—	—	—
Unigene2670_yong_A	817	gi|356470637|gb|AET09964.1|	1.00E-154	*P. xylostella*
Unigene2920_yong_A	1,220	gi|354463161|gb|AER25355.1|	0	*P. xylostella*
Unigene33701_yong_A	313	gi|356470637|gb|AET09964.1|	2.00E-56	*P. xylostella*
Unigene5667_yong_A	2,595	gi|356470637|gb|AET09964.1|	0	*P. xylostella*

### Digital gene expression (DGE) library sequencing

To analyze the gene expression variations during the occurrence and development of CR in *P. xylostella*, four DGE libraries of four *P. xylostella* populations, including a susceptible strain (SS), low level resistance strain (GXA), moderate resistance strain (LZA) and high resistance strain (HZA), were sequenced using RNA-Seq. After removing the low quality reads, the total number of clean reads per library ranged from 8.4 to 8.9 million. Among these clean reads, 6.2 to 7.2 million were mapped to unigenes (Table 4).

**Table 4:** Alignment statistics of the RNA-Seq analysis.

Map to Gene	SS	LZA	GXA	HZA
Total reads	8,871,243	8,500,303	8,495,476	8,693,500
Total base pairs	434,690,907	416,514,847	416,278,324	425,981,500
Total mapped reads	7,204,381	6,387,754	6,294,232	6,536,234
Perfect match	5,023,111	3,500,612	3,435,059	3,482,123
< = 2 bp mismatch	2,181,270	2,887,142	2,859,173	3,054,111
Unique match	6,237,793	5,352,278	5,485,429	5,842,072
Multi-position match	966,588	1,035,476	808,803	694,162
Total unmapped reads	1,666,862	2,112,549	2,201,244	2,157,266

**Figure 3:**
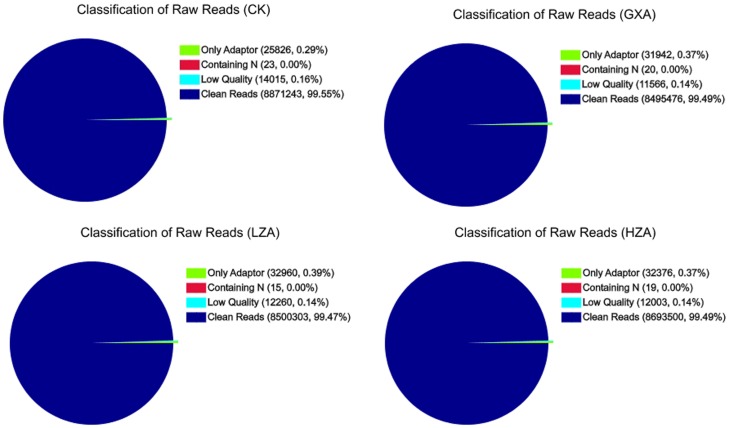
Evaluation of sequence quality for the four resistance levels of *P. xylostella*. CK: SS Strain; LZA: LLR strain; GXA: MR strain; HZA: HR strain.

Clean reads are the data after the raw dirty reads are removed. The percentages of clean reads in the four libraries were all over 99%, and the low quality reads ranged from 0.14% to 0.15%, reflecting the high quality of the sequence (Fig. 3). The gene coverage statistics indicate that 23%, 15%, 19% and 18% of the genes were covered between 90–100% in the SS, LZA, GXA and HZA, respectively, and that fewer than 2% of the genes were covered by 0∼10% (Fig. S2).

### Comparison of the gene expression profiles among the different chlorantraniliprole-resistant *P. xylostella* populations

To identify the genes that are associated with the CR levels in *P. xylostella*, the differential gene expressions of populations with different CR levels were studied by variation analysis using a strict algorithm [25]. The populations with different CR levels produced different LC50 values and different gene expression patterns compared with the SS population. For example, the LC50 for the GXA, LZA and HZA populations were 1.35, 7.97, and 402.49 mg/L, respectively, which are 5.87-, 34.65- and 1749.96-fold compared to the SS population, respectively. These three resistant populations have 43,143, 43,147 and 42,941 genes with differential expression levels, respectively (Fig. S3).

**Figure 4:**
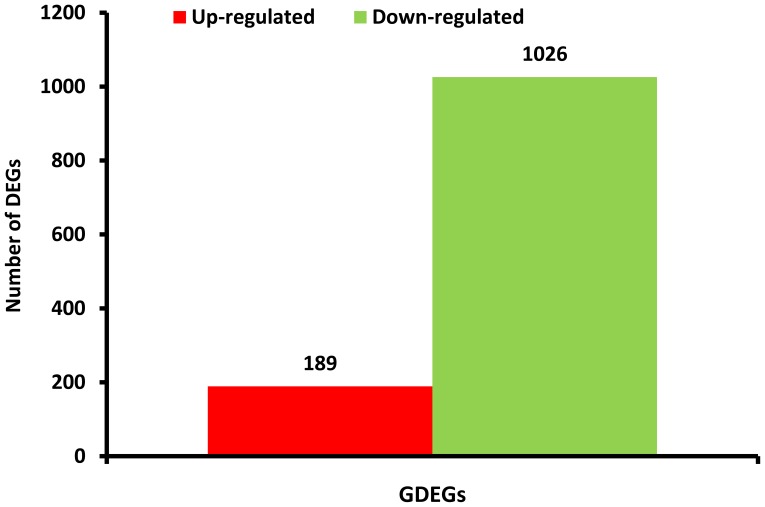
Summary of differently expressed genes in each pariwise comparison. GDEGs: Gradient differentially expressed genes, HZA-VS-SS.LZA-VS-SS. GXA-VS-SS

For exploring the genes related to chlorantraniliprole resistance development, the gradient differentially expressed genes (GDEGs) in the SS, GXA, LZA and HZA populations were analyzed in detail. A total of 1,215 gradient differentially expressed genes up-regulated or down-regulated with the change in the gradient level of *P. xylostella* resistance to chlorantraniliprole were obtained. There were 189 genes up-regulated with the increase in the resistance level, whereas 1,026 genes were down-regulated with the increase in the resistance level to chlorantraniliprole (Fig. 4, Table S3). Of these gradient differentially expressed genes, ryanodine receptors, calcium ATPase, flocculins, and connectins are involved in calcium signaling, vascular smooth muscle contraction and cardiac muscle contraction pathways, whereas cCaE, GSTe, cytochrome P450, and PPO are responsible for the metabolic pathways, such as drug and xenobiotic metabolism, which are predominantly triggered by cytochrome P450. Those gradient differentially expressed genes that are involved in different pathways may be associated with the resistance of *P. xylostella* to chlorantraniliprole.

### Analyzing the genes related to the resistance molecular mechanisms of *P. xylostella* to chlorantraniliprole

**Figure 5:**
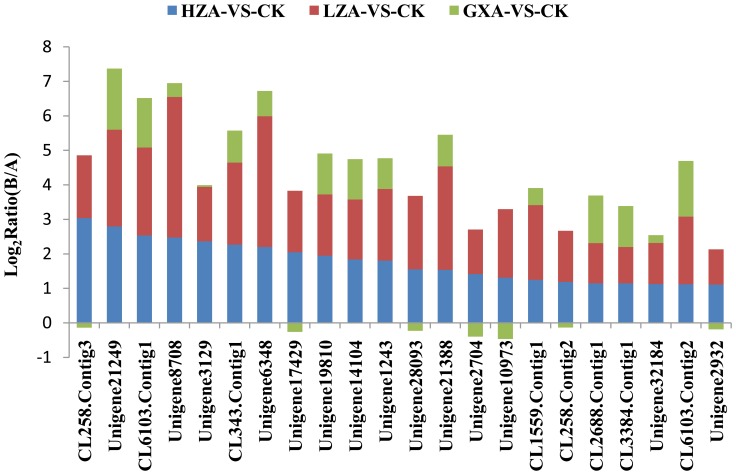
The expression trend and level of each metabolic gene for the gradient differentially expressed genes.

**Figure 6:**
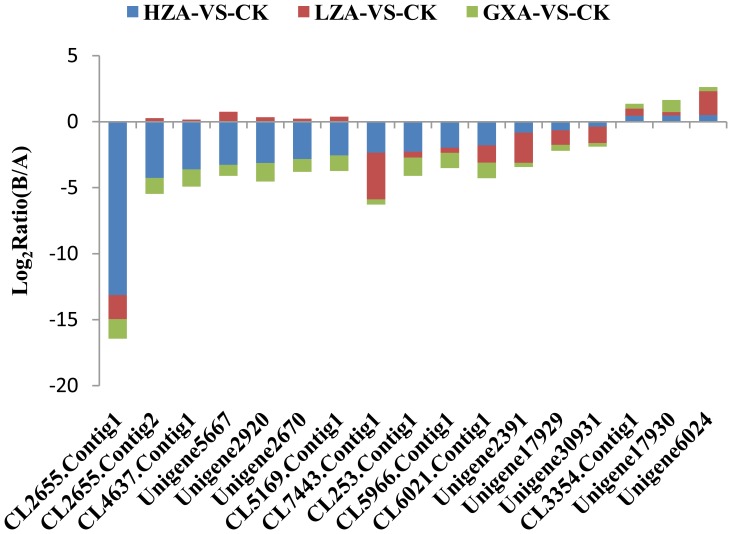
The expression trend and level of ryanodine receptor genes for the gradient differentially expressed genes.

**Figure 7:**
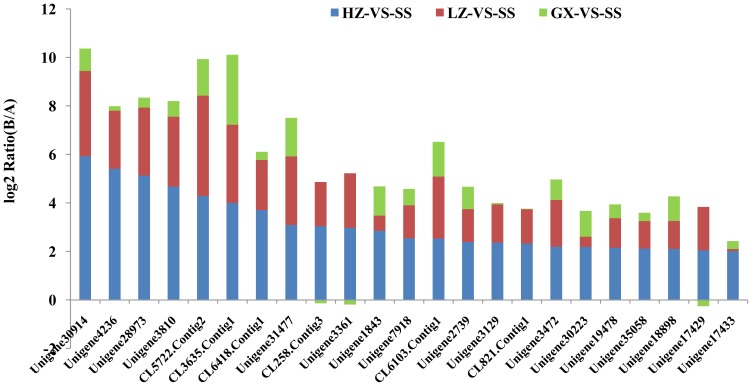
The expression trend and level of genes involved in the metabolic pathways. The metabolic pathways include metabolic pathways, drug metabolism-cytochrome P450, metabolism of xenobiotics by cytochrome P450 and drug metabolism-other enzymes.

The detoxification metabolism and modification of insecticide target proteins are the primary factors that induce insecticide resistance in insects [19]. Based on the GDEGs, a total of 22 metabolic detoxification enzyme unigenes (including 19 P450 unigenes and 3 GST unigenes) and 17 ryanodine receptor unigenes that are gradient differentially expressed with the increasing resistance of *P. xylostella* to chlorantraniliprole were identified. With this particular analysis, we discovered that in GXA populations, most of the metabolic detoxification enzyme genes were slightly up-regulated and, in the LZA populations, were significantly up-regulated. However, there was no significant difference between the LZA populations and HZA populations. For example, unigene21249_yong_A had a ‘log2 Ratio (GXA/SS)’ of 1.77; however, the ‘log2 Ratio (LZA/SS)’ and ‘log2 Ratio (HZA/SS)’ displayed no obvious change from 2.81 to 2.79 (Fig. 5). In contrast, the ryanodine receptor genes were slightly down-regulated in the low- and medium-level resistance populations GXA and LZA, respectively, whereas their levels were significantly down-regulated in the high-level resistance population HZA, such as the ‘log2 Ratio (GXA/SS)’ and ‘log2 Ratio (LZA/SS)’of unigene CL2655. Contig1_yong_A was -1.49 and -1.81, and the difference was not significant. However, the ‘log2 Ratio (HZA/SS)’ decreased to -13.13, with the down-regulation being very significant (Fig. 6). Four prophenoloxidase unigenes (unigene30914_yong_A, unigene4236_yong_A, unigene28973_yong_A, and unigene3810_yong_A) were up-regulated significantly with the raising of the chlorantraniliprole resistance level. For example, the ‘log2 Ratio (GXA/SS)’ of unigene30914_yong_A was 0.92, and the ‘log2 Ratio (LZA/SS)’ and ‘log2 Ratio (HZA/SS)’ were 3.52 and 5.93, respectively, the difference being very significant (Fig. 7), suggesting that prophenoloxidase plays a role in defending against the chlorantraniliprole pesticide.

From the above results, we deduced that at the initial point of chlorantraniliprole resistance development, the expression of the genes encoding for the detoxification enzymes were up-regulated, whereas during later periods, the targets of chlorantraniliprole were down-regulated. This result suggests that the up-regulation of detoxification genes, such as P450, GSTs and prophenoloxidase, and the down-regulation of ryanodine receptors, such as the chlorantraniliprole-binding target, are the main factors leading to the development of chlorantraniliprole resistance.

**Figure 8:**
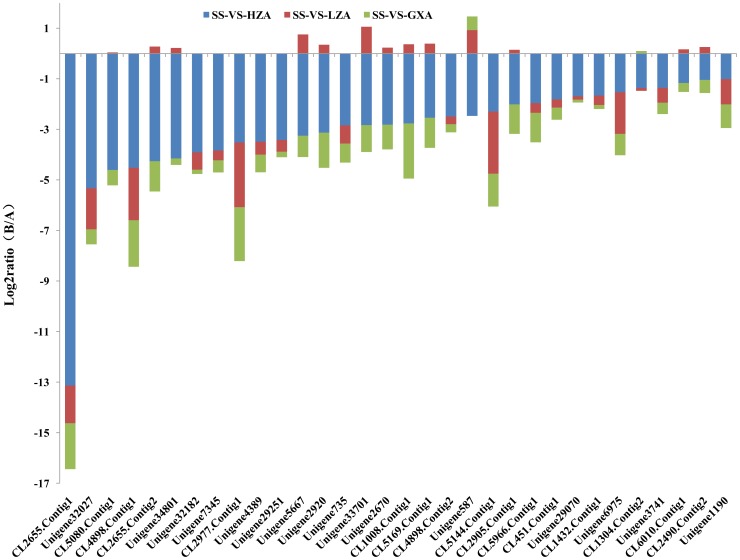
The expression trend and level of genes involved in the calcium signaling pathways of *P. xylostella*.

**Figure 9:**
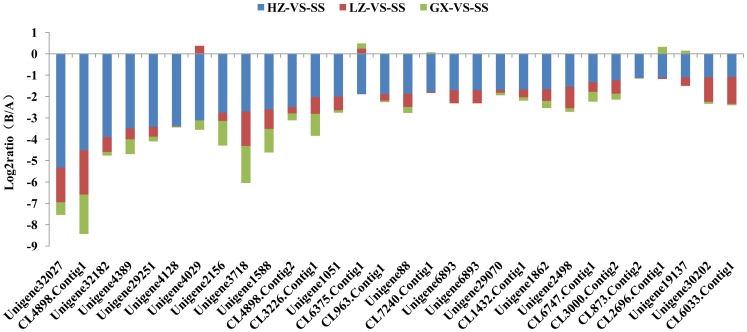
The expression trend and level of genes involved in the muscle control pathways of *P. xylostella.* The muscle contraction pathways include vascular smooth muscle contraction and cardiac muscle contraction.

Chlorantraniliprole can act on ryanodine receptors, which play a vital role in calcium signaling pathways and muscle control pathways composed of vascular smooth muscle contraction and cardiac muscle contraction, and cause the continuing loss of calcium, muscle weakness, and eventually kill the pests [14]. In total, 32 GDEGs genes were annotated from the calcium signaling pathways, including the ryanodine receptor CL2655, Contig1_yong_A, and flocculin unigene32027_yong_A. In addition, 31 GDEGs genes are involved in vascular smooth muscle contraction and cardiac muscle contraction, such as flocculin unigene32027_yong_A. All of the GDEG genes involved in calcium signaling pathways and muscle control pathways were down-regulated with the increase in chlorantraniliprole resistance (Figs. 8, 9). The down-regulation of genes involved in calcium signaling pathways and muscle control pathways may lead to decreases in chlorantraniliprole-binding target proteins, which may be another factor related to the resistance of *P. xylostella* against chlorantraniliprole.

### Quantitative RT-PCR validation of the gene expression profile

**Figure 10:**
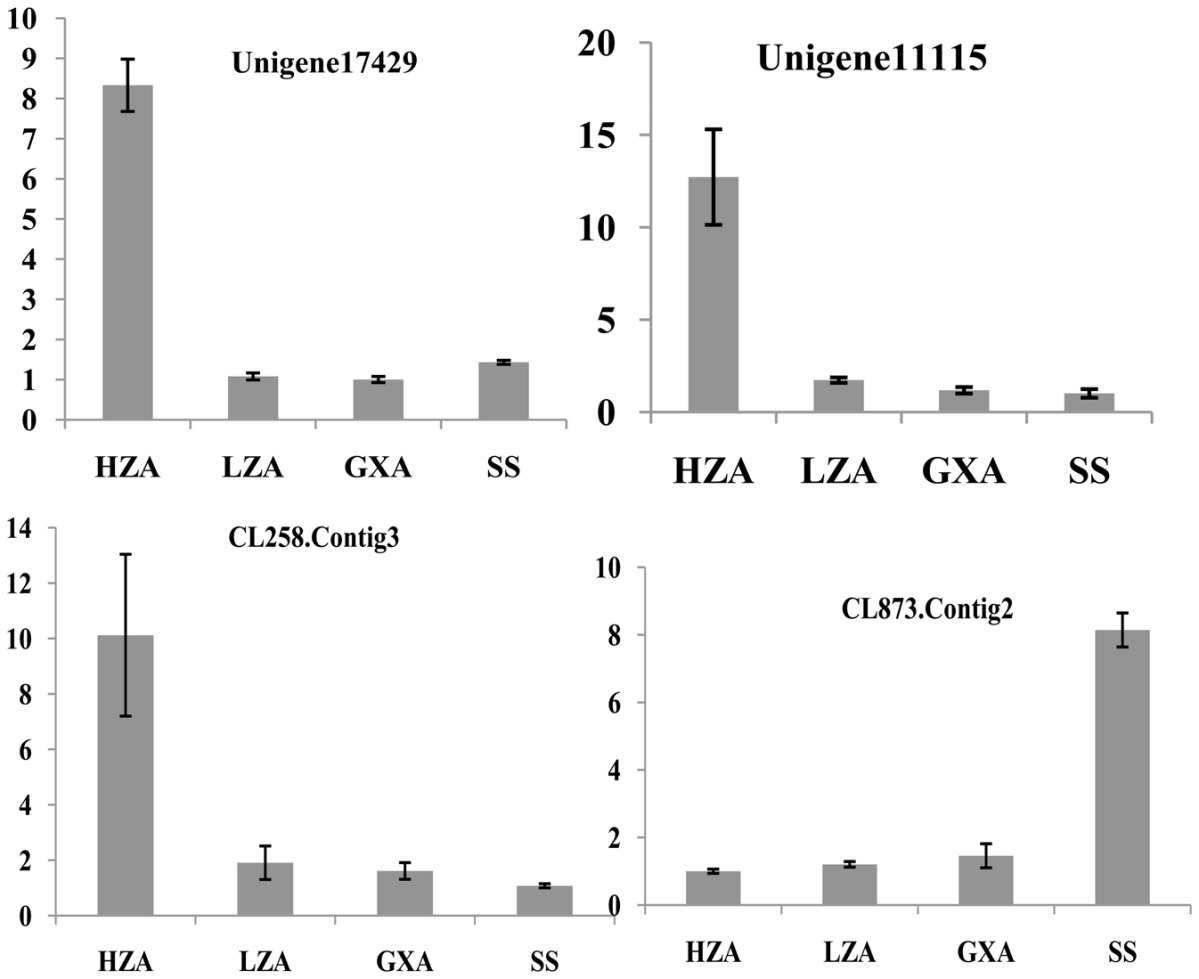
qRT-PCR analysis of four selected genes from *P. xylostella* that showed differential expression with differential resistance levels, as based on RNA-seq analysis. Error bars indicate the average deviations of the three replicates.

To validate the findings from the sequencing data, four differentially expressed genes were selected at random from the gradient differentially expressed genes by qRT-PCR analysis. The results confirmed that the expression patterns of these target genes had similar trends with the data obtained from the RNA-seq (Fig. 10).

## Discussion

Insecticide resistance is a complex biological event and is related to mutations affecting insecticide target proteins, metabolism and other cellular processes [19]. Over the past years, insecticide detoxification metabolism- and target mutation-mediated insecticide resistances have been most commonly studied and described by means of different molecular tools [26], [27], [28]. However, the specific metabolic pathways involved in the development of insecticide resistances are not yet completely clear. Next-generation sequencing technologies have promoted research on insecticide resistance in insects with unknown genomes or incomplete protein databases and have helped us to obtain a more comprehensive knowledge of insecticide resistance mechanisms. A total of 211 metabolism genes and target genes related to general insecticide resistance, such as P450 and cytochrome b, have been identified from global transcriptomes between acaricide-resistant strains and susceptible strains of *P. citri* using next-generation sequencing technology [22]. The transcriptomes of *P. xylostella* have also been *de novo* assembled and annotated using short reads generated by Illumina sequencing from different developmental stages and from insecticide-resistant strains [21], [35]. Based on these transcriptomes, the differentially expressed unigenes reveal that the enriched GOs and biological pathways are related to specific developmental stages and insecticide resistance. These results provide a valuable genomic resource for further understanding the molecular basis of resistance mechanisms. In our study, the transcriptome of a *P. xylostella* susceptible strain and four digital gene expression profiles of 3^rd^ instar larvae were sequenced. The gradient differentially expressed genes provide us with effective genetic information for elucidating the CR molecular mechanisms in *P. xylostella*.

Insecticide detoxication occurs in all insects, and a number of enzymes with insecticide detoxication mechanisms, encoded for by members of the P450, GST and COE multi-gene families, have been identified through insecticide resistance studies [26]. Detoxification by enhancing monooxygenase activities is one of the major mechanisms of pyrethroid resistance, along with reducing the sensitivity of target sites associated with the nervous system [29], [30]. Some P450 genes, such as *CYP6BF1*, *CYP6BG1* and *CYP9G2*, have been isolated from *P. xylostella* [31], [32], [33]. Over- expressing *CYP6BG1* results in permethrin resistance, which was evaluated by RNA interference-mediated gene silencing (RNAi) tests [34]. Many *P. xylostella* GST isozyme genes (such as *PxGSTs* and *PxGSTe*), which are involved in resistance to some organophosphorus insecticides, have been cloned and heterologously expressed [29], [36]. In addition, studies on inhibitors of metabolic enzymes have suggested that enhanced enzymatic detoxification may play a role in the CR of the beet armyworm (*Spodoptera exigua* Hübner) and the obliquebanded leafroller (*Choristoneura rosaceana* Harris) [37], [38]. Mutating ryanodine receptors is one of the factors leading to the development of CR in *P. xylostella* [39]. However, the production of insecticide resistance is complex. Therefore, possible molecular mechanisms of CR in *P. xylostella* will require more information for elucidation. In this study, 19 P450 genes and 3 GST genes that gradient up-regulated the level of resistance from low to high were identified, for example, the unigene CL258. Contig3-yong-A was annotated to *CYP6BG1*, which is known to be one of the reasons for *P. xylostella* resistance against pemethrin [34]. However, wheather *CYP6BG1* is responsible for CR is unkown. Although these genes’ functions in the mechanisms of CR in *P. xylostella* have not yet been analyzed, their expression patterns with increased levels of resistance indicate that they may be involved in the CR of *P. xylostella* at its initial phase.

The expression and structural transformation of insecticide target proteins are the major mechanisms of insecticide resistance [40]. Studying the RyR is one of the key areas for understanding the CR mechanism. The full-length cDNA encoding for a ryanodine receptor has been cloned from *P. xylostella*, and its mRNA expression profile was characterized in the head, thorax, and abdomen of fourth instar larvae [41]. A target site mutation in the C-terminal membrane-spanning domain of the RyR was found in *P. xylostella* collected from the Philippines and Thailand, which were found to be over 200-fold more resistant to chlorantraniliprole compared to susceptible strains. This mutation may confer at least part of the observed resistance phenotype [39]. In our study, slight down-regulations in the low- and medium-level resistance populations GXA and LZA and significant down-regulations in the high-level resistance population HZA were observed, such as CL2655.Contig1_yong_A. Our results suggest that during later periods of chlorantraniliprole resistance development, the down-regulation of ryanodine receptors can lead to decreased chlorantraniliprole target binding, resulting in the resistance of *P. xylostella* to chlorantraniliprole.

Insecticide metabolism involves a multistep pathway that almost certainly contains multiple enzymes. This paradigm arises from the mammalian drug metabolism literature; however, metabolic pathways involving insect resistance to insecticides have been rarely reported [19]. Therefore, finding the underlying metabolic pathways and identifying the precise enzymes involved in these processes in insects are challenging. Insecticide resistance is a genetic phenomenon [42]. For years, our understanding of the available evolutionary options for insecticide resistance has been superficial because of the limitations of genetic techniques. With the application of high-throughput sequencing platforms in resistance research, more and more metabolic pathways related to insecticide resistance will be discovered and studied. The metabolic pathways involved in studying acaricide and chlorpyrifos resistances in the citrus red mite and *P. xylostella* have been reported [21], [22], and these results will help us to understand the development of insect resistance to insecticides and to study specific approaches that could be taken for resistance management. In this study, a KEGG analysis showed that the gene expression levels in metabolic pathways, such as drug metabolism-cytochrome P450, metabolism of xenobiotics by cytochrome P450 and drug metabolism-other enzymes, were up-regulated with the increasing CR level. Some genes were annotated as involved in more than one metabolic pathway, such as gene CL258. Contig3_yong_A has been annotated in the following metabolic pathways (no map in the KEGG database): drug metabolism-other enzymes, drug metabolism-cytochrome p450, metabolism of xenobiotics by cytochrome P450, linoleic acid metabolism and bile secretion. In the present study, other genes involved in calcium signaling pathways and muscle control pathways, such as the calcium ATPase gene CL2977. Contig1_yong_A, flocculin gene unigene32027-yong-A and connectin gene CL4898.contig-yong-A, were also found to be down-regulated as the CR level increases. From the above results, it was deduced that pathways, such as metabolic pathways, calcium signaling pathways and muscle control pathways, involving multiple genes play a dominant role in the process of CR generation.

In our study, the molecular functions of individual *P. xylostella* genes and the related signal transduction and metobolic pathways remain largely unclear; however, the present transcriptome and DGE data provide valuable information regarding the development of chlorantraniliprole resistance, which could facilitate further investigations of the detailed resistance mechanisms of *P. xylostella* against chlorantraniliprole.

## Materials and Methods

### Insect rearing and sampling

A susceptible *P. xylostella* strain (SS) was collected in July 2002 from vegetable fields (Guangdong, China) and was maintained without exposure to insecticides for nine years. Three CR strains were collected from cabbage fields in Liuzhou, Guangxi Province (GXA), Lianzhou (LZA) and Huizhou (HZA), Guangdong Province, respectively. The field-collected strains were maintained on cabbage in the laboratory for 4 generations and selected using chlorantraniliprole each generation to select against sensitive individuals and to remove the influence of other field factors. The rearing conditions were maintained at 25±2°C, 70–80% RH and a photoperiod of 16-h light/8-h dark. The median lethal concentrations (LC_50_) of each sample to chlorantraniliprole were 0.226 mg/L (SS), 1.35 mg/L (GXA), 7.97 mg/L (LZA) and 402.49 mg/L (HZA), respectively, and the resistance ratios (LC_50_ of a resistant strain/LC_50_ of the SS strain) were 5.87, 34.65 and 1749.96, indicating a low level resistance (GXA), moderate resistance (LZA) and high resistance (HZA), respectively. However, the resistance levels of the three population to other common insecticides were similar (Table S4). Eight samples were analyzed, including newly laid *P. xylostella* eggs, 1^st^, 2^nd^, 3^rd^ and 4^th^ instar larvae, pupae, and adults of the SS, and the third instar larvae of the GXA, LZA and HZA.

### cDNA library construction and Illumina sequencing

Total RNA was extracted from the whole bodies of each sample and treated with fragmentation buffers for interrupting mRNA to short fragments. Taking these short fragments as templates, random hexamer primers were used to synthesize the first-strand cDNA. The second-strand cDNA was synthesized using buffer, dNTPs, RNaseH and DNA polymerase I. Short fragments were purified using a QIAquick PCR extraction kit and resolved with EB buffer for end repair and A tailing (Omega Bio-Tek, Inc. 400 Pinnacle Way, Suite 450 Norcross, GA 30071, USA). The short fragments were then connected with sequencing adapters. After agarose gel electrophoresis, the suitable fragments were selected for the PCR amplification as templates. Finally, the library was sequenced using Illumina HiSeq™ 2000.

### Assembly and functional annotation

The raw reads were cleaned up by removing the reads with adaptors, unknown nucleotides larger than 5% and low quality reads. Deep sequencing-based transcriptome profiling was performed using a short read assembling program – Trinity [43]. Trinity first combines reads of a certain length that overlap to form longer fragments, which are called contigs. The reads were then mapped back to contigs with paired-end reads. The program is then able to detect contigs from the same transcript as well as the distances between these contigs. Finally, Trinity connects the contigs and obtains sequences that cannot be extended on either end. Such sequences are defined as unigenes. After clustering, the unigenes are divided into two classes: clusters, with the prefix CL, and singletons, having the prefix unigene. In the final step, a blastx alignment (E-value <0.00001) between unigenes and protein databases such as nr, Swiss-Prot, KEGG and COG was performed, and the best alignment results were used to decide the sequence direction of the unigenes. If the results from the different databases conflicted with each other, a priority order of nr, Swiss-Prot, KEGG and COG was followed when deciding the sequence direction of the unigenes. When a unigene happened to be unaligned to none of the above databases, a software known as ESTS was introduced to decide its sequence direction [44]. With the nr annotation, we used the Blast2GO program to obtain the GO annotation for the unigenes [45]. After obtaining the GO annotation for every unigene, we used the WEGO software to perform a GO functional classification for all unigenes and to understand the distribution of the gene functions of the species from the macro level [46].

### Differential expression of the uingenes

The gene expression level was calculated by using the RPKM method (reads per kb per million reads) [47]. This analysis finds genes that have different expression levels among samples and then performs a GO function analysis and KEGG pathway analysis [48]. The *P*-value corresponds to the differential gene expression test. FDR (false discovery rate) is a method to determine the threshold for a *P*-value in multiple tests. We assume that we picked out R differentially expressed genes in which S genes actually show differential expressions and that the other V genes are false positives. If we decide that the error ratio "Q  =  V/R" must stay below a cutoff, we should preset the FDR to a number no larger than 0.01 [49]. We used "FDR ≤ 0.001 and the absolute value of log2Ratio ≥ 1" as the threshold to judge the significance of the gene expression difference. More stringent criteria with smaller FDR and larger fold-change values can be used to identify DEGs.

### Gene ontology analysis of DEG

The analysis first maps all DEGs to GO terms in the database by virtue of calculating gene numbers for every term, followed by an ultra-geometric test to find significantly enriched GO terms in DEGs compared to the transcriptome background. The formula for this calculation was:
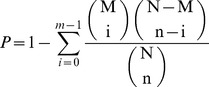



N is the number of all genes with a GO annotation, n is the number of DEGs in N, M is the number of all genes that are annotated to certain GO terms, and m is the number of DEGs in M. The calculated *P*-value goes through Bonferroni correction, taking a corrected *P*-value ≤0.05 as a threshold. GO terms fulfilling this condition are defined as significantly enriched GO terms in DEGs. This analysis is able to recognize the main biological functions that the DEGs exercise.

### Pathway enrichment analysis of DEG

Genes typically interact with each other to play roles in certain biological processes. Pathway-based analyses help us to further understand the biological functions of the genes. KEGG is the major public pathway-related database. Pathway enrichment analyses identify significantly enriched metabolic pathways or signal transduction pathways in DEGs compared with the whole genome background. The calculating formula was the same as that in the GO analysis. Here N is the number of all genes with a KEGG annotation, n is the number of DEGs in N, M is the number of all genes annotated to specific pathways, and m is number of DEGs in M.

### Quantitative RT-PCR (qRT-PCR) validation of the deep-sequencing data

The deep-sequencing data were validated by quantitative RT-PCR technique using the same RNA samples that were used for transcriptome and DGE profiling. First strand cDNA was synthesized from 1 µg of RNA using M-MLV reverse transcriptase (New England Bio-Labs). The qRT-PCR reaction consisted of 1 µL of diluted cDNA, 10 µL of Platinum SYBR Green Super-Mix-UDG with ROX (Invitrogen), 0.5 µM of each primer (Table 5) and 8 µL ddH_2_O (20 µL total volume). The reactions were performed in triplicate in a Rotor-Gene thermal cycler (BIORAD) under the following conditions: 4 min of initial preheating at 94°C; 30 cycles of 94°C for 30 s, 62°C for 30 s and 72°C for 30 s; and a final elongation step at 72°C for 6 min. Melting curves for each sample were analyzed to check the specificity of the amplification. Gene copy numbers were calculated using the Rotor-Gene software. A reaction performed without a DNA sample (ddH_2_O substitute) was used as the negative control, and an endogenous actin reference gene was used for normalization.

**Table 5:** The primer sequence used for each gene for the quantitative RT-PCR.

Gene	Forward primer	Reverse primer
CL258	GCTCATCACCACCAAGGACT	CCTGAGCACCTTCCATTTGT
Unigene17429	CCCTCGGTGAATATGAGGAA	GTACCCCAAGGACGTGAAGA
Unigene11115	TGAAAGAGACGGTGCTGATG	GAGCGAGAACATCCTTTTGC
CL873	CGAGCGACCTCATCGTATTT	AGAACAAGTCGCTGGCTGAT
Actin	TGGCACCACACCTTCTAC	CATGATCTGGGTCATCTTCT

### Ethics Statement

No specific permits were required for the described field studies. No specific permissions were required for these locations. We confirm that the location is not privately owned or protected in any way. We confirm that the field studies did not involve endangered or protected species.

## Supporting Information

Figure S1 **Characteristics of the homology search for the Illumina sequences against the nr database.** (A) E-value distribution of BLAST hits for each unique sequence with a cut-off E-value of 1.0E-5. (B) Similarity distribution of the top BLAST hits for each sequence. (C) The species distribution is shown as a percentage of the total homologous sequences with an E-value of at least 1.0E-5.
(TIF)

Figure S2 **Distribution for the gene coverage at each level of resistance for the** ***P. xylostella*** **library.** CK: SS Strain; LZA: LLR strain; GXA: MR strain; HZA: HR strain
(TIF)

Figure S3 **Differences in the gene expression profiles between each resistant strain and the susceptible strain.**
(TIF)

Table S1 **Top hits obtained by BLASTX for the unigenes.**
(XLS)

Table S2 **KEGG annotation of unigenes.**
(XLS)

Table S3 **The gradient differentially expressed genes (GDEGs) following the level of resistance from low to high.**
(XLS)

Table S4 **The LC_50_ of each population to chlorantraniliprole and other common insecticides.** RR: Resistance ratio  = LC50 of a peld pop/LC50 of the Roth strain; LC50: mg/liter, 95% FL; Resistance level: low level resistance, 0<RR<10; moderate resistance, 10<RR<100; high resistance, RR>100.
(DOC)
