# Supplementary figures and images for: Transcriptome Analysis of Chlorantraniliprole Resistance Development in the Diamondback Moth Plutella xylostella
Source: PLoS One. 2013 Aug 20;8(8):e72314. doi: 10.1371/journal.pone.0072314 (PMC3748044; doi:10.1371/journal.pone.0072314)

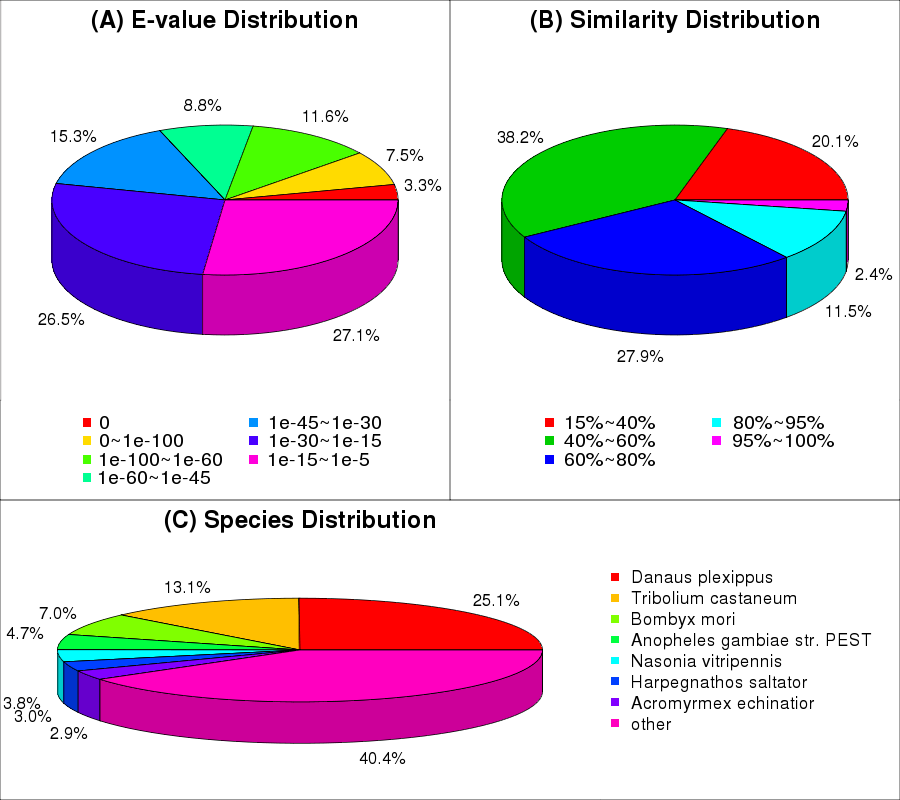

Supplement: Figure S1 — Characteristics of the homology search for the Illumina sequences against the nr database. (A) E-value distribution of BLAST hits for each unique sequence with a cut-off E-value of 1.0E-5. (B) Similarity distribution of the top BLAST hits for each sequence. (C) The species distribution is shown as a percentage of the total homologous sequences with an E-value of at least 1.0E-5. (TIF) [file pone.0072314.s001.tif]

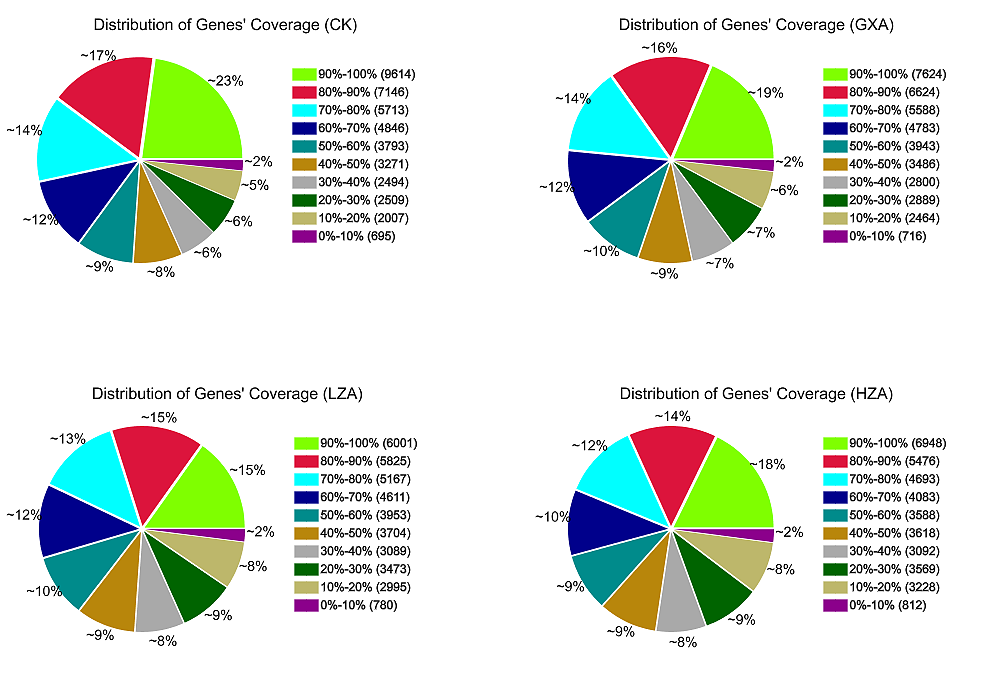

Supplement: Figure S2 — Distribution for the gene coverage at each level of resistance for the P. xylostella library. CK: SS Strain; LZA: LLR strain; GXA: MR strain; HZA: HR strain (TIF) [file pone.0072314.s002.tif]

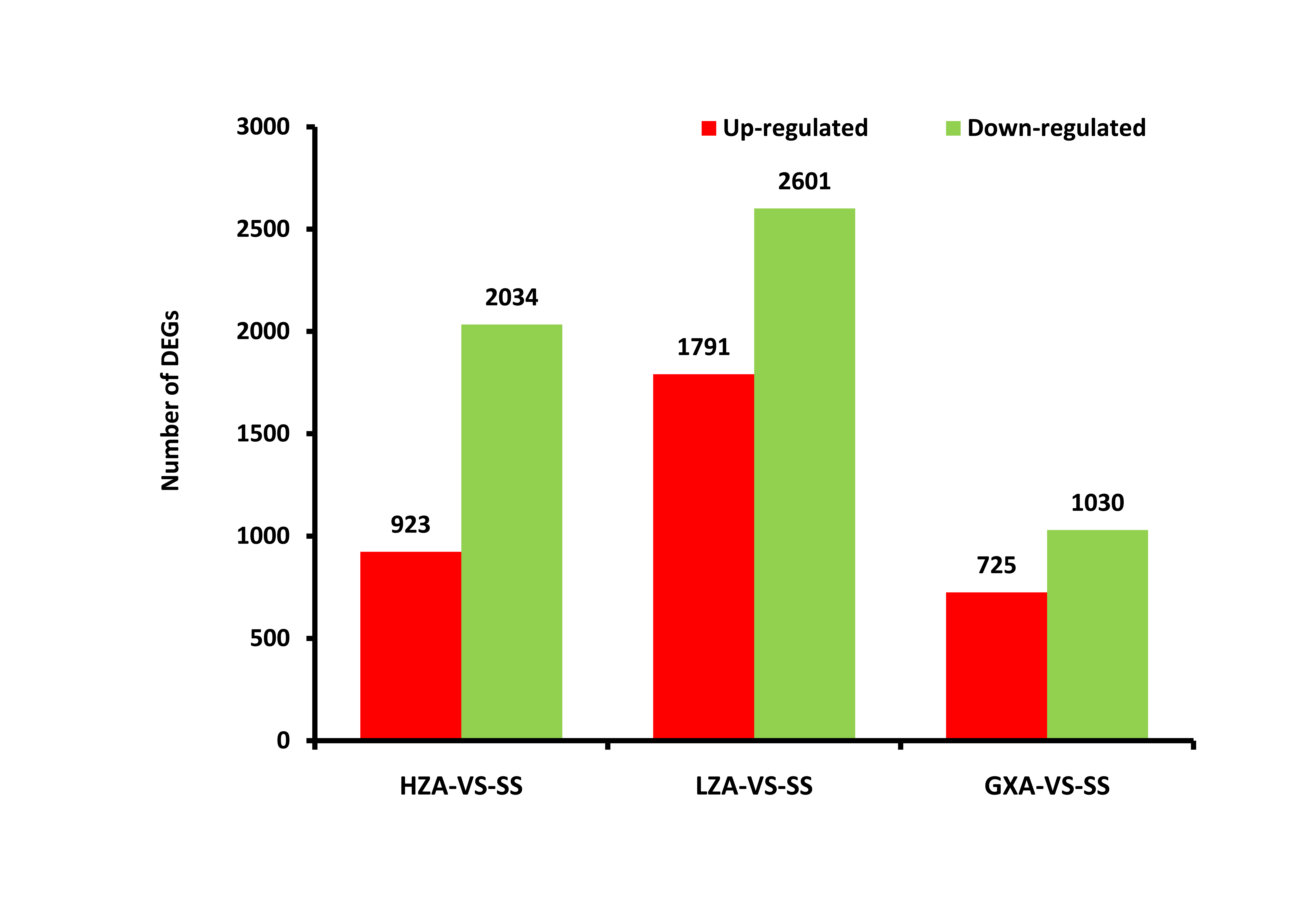

Supplement: Figure S3 — Differences in the gene expression profiles between each resistant strain and the susceptible strain. (TIF) [file pone.0072314.s003.tif]
