# Supplementary material for: Transcriptome Analysis of Chlorantraniliprole Resistance Development in the Diamondback Moth Plutella xylostella
Source: PLoS One. 2013 Aug 20;8(8):e72314. doi: 10.1371/journal.pone.0072314 (PMC3748044; doi:10.1371/journal.pone.0072314)
Supplement: Table S4 — The LC50 of each population to chlorantraniliprole and other common insecticides. RR: Resistance ratio = LC50 of a peld pop/LC50 of the Roth strain; LC50: mg/liter, 95% FL; Resistance level: low level resistance, 0<RR<10; moderate resistance, 10<RR<100; high resistance, RR>100. (DOC) [file pone.0072314.s007.doc]

Table S4 The LC_50_ of each population to chlorantraniliprole and other common insecticides

| Population | Chlorantraniliprole | | Spinosad | | Bt | | Abamectin | | Chlorfluazuron | | Diafenthiuron | |
| --- | --- | --- | --- | --- | --- | --- | --- | --- | --- | --- | --- | --- |
|  | LC_50_ | RR | LC_50_ | RR | LC_50_ | RR | LC_50_ | RR | LC_50_ | RR | LC_50_ | RR |
| SS | 0.226 |  | 0.12 |  | 0.26 |  | 0.02 |  | 0.33 |  | 21.387 |  |
| GXA | 1.35 | 5.87 | 0.38 | 3.17 | 0.27 | 1.04 | 29.19 | 1459.5 | 1.38 | 4.18 | 51.74 | 2.42 |
| LZA | 7.97 | 34.65 | 0.06 | 0.50 | 0.24 | 0.92 | 30.0 | 1500.0 | 0.37 | 1.12 | 2.11 | 0.10 |
| HZA | 402.49 | 1749.96 | 3.53 | 29.33 | 1.50 | 5.77 | 282.80 | 14140.0 | 2.89 | 8.76 | 116.32 | 5.44 |

RR: Resistance ratio =LC50 of a Þeld pop/LC50 of the Roth strain;

LC50: mg/liter, 95% FL;

Resistance Level: low level resistance, 0<RR<10; moderate resistance, 10<RR<100; high resistance, RR>100
